# Characterization of polysaccharides from *Tetrastigma hemsleyanum* Diels et Gilg Roots and their effects on antioxidant activity and H_2_O_2_-induced oxidative damage in RAW 264.7 cells

**DOI:** 10.1186/s13065-021-00738-1

**Published:** 2021-02-05

**Authors:** Qi Huang, Wen He, Ilkhomjon Khudoyberdiev, Chun-Lin Ye

**Affiliations:** School of Biological and Chemical Engineering, Zhejiang University of Science and Technology, Hangzhou, 310023 P. R. China

**Keywords:** Antioxidant activity, Polysaccharides, Structural characterization, RAW 264.7 cells, *Tetrastigma hemsleyanum*

## Abstract

This work presents an investigation on the composition and structure of polysaccharides from the roots of *Tetrastigma hemsleyanum* (THP) and its associated antioxidant activity. It further explores the protective effect of THP on RAW264.7 cells against cytotoxicity induced by H_2_O_2_. Ion chromatography (IC) revealed that THP contained glucose, arabinose, mannose, glucuronic acid, galactose and galacturonic acid, in different molar ratios. Furthermore, gel permeation chromatography-refractive index-multiangle laser light scattering (GPC-RI-MALS) was employed to deduce the relative molecular mass (M_w_) of the polysaccharide, which was 177.1 ± 1.8 kDa. Fourier transform infrared spectroscopy (FT-IR) and Congo red binding assay highlighted that the THP had a steady α-triple helix conformation. Similarly, assays of antioxidant activity disclosed that THP had reasonable concentration-dependent hydroxyl radical and superoxide radical scavenging activities, peroxidation inhibition ability and ferrous ion chelating potency, in addition to a significant 1,1-diphenyl-2-picrylhydrazyl radical scavenging capacity. Moreover, THP could protect RAW264.7 cells against H_2_O_2_-induced cytotoxicity by decreasing intracellular ROS levels, reducing catalase (CAT) and superoxide dismutase (SOD) activities, increasing lactate dehydrogenase (LDH) activity and increment in malondialdehyde (MDA) level. Data retrieved from the in vitro models explicitly established the antioxidant capability of polysaccharides from *T. hemsleyanum* root extracts.

## Introduction

Bio-medicinal developments keep unfolding the crucial roles played by free radicals in living organisms. The discovery of more natural, safe and effective antioxidants has been a hot topic over the recent years, as they can defend human bodies from free radicals, at the same time retarding the advancement of numerous chronic diseases. *Tetrastigma hemsleyanum* is a naturally occurring herbal plant which prevalently grows in moist, shady hillsides and valleys, and belongs to the grape family (*Vitaceae*). It is mainly distributed around the central, eastern, southern and southwestern provinces of China [1]. As a customary Chinese medicine, it is mainly used to treat high fever, pneumonia, rheumatism, infantile febrile convulsion, asthma, hepatitis, sore throat, and menstrual disorders [2]. Earlier reports have indicated that the roots of *T. hemsleyanum* contain various phytochemicals which includes polysaccharides [3], flavonoids [4], lipids [5] and phytosterols [6]. In addition, research in modern pharmacology has indicated that these extracts have some immune-regulatory action [7], antitumor effect [8, 9] and largely antioxidant activity [10]. However, advancements in biomedicine have revealed that the pathological pathways of several ailments are attributed by the imbalance of free radical metabolism and lipid peroxidation. Many diseases are caused by excessive oxygen free radicals which oxidize and destroy normal cells [11, 12]. Therefore, in order to balance the oxidative stress, exogenous antioxidants are constantly needed to sustain an adequate level.

For the past two decades, the search for safe and functional antioxidants has redirected to natural products, owing to some carcinogenic effects found in synthetic antioxidants [13, 14]. Polysaccharides are macromolecular compounds broadly distributed in plants and animals, with numerous fundamental biological activities such as regulation of immunity [15], antifungal effect [16], antitumor effect [17], antioxidant activity [18], and antiviral activity [19]. Recently, antioxidant activities of polysaccharides and their intrinsic effects have been comprehensively studied both at home and abroad [20, 21]. The optimum extraction conditions of the polysaccharides from the roots of *T. hemsleyanum* (THP), which are already known to contain a great number of polysaccharides, were determined by response surface methodology [3]. Nonetheless, to the best of our knowledge, little research has been reported on the chemical structural elucidation and antioxidant activity of polysaccharide from the roots of *T. hemsleyanum*. Therefore, the purpose of the present study was to investigate the composition, antioxidant ability and the protective effect of THP on RAW 264.7 cells against cytotoxicity induced by H_2_O_2_.

## Materials and methods

### Materials

Dried roots of *Tetrastigma hemsleyanum* were bought from Lishui, in Zhejiang Province, China. Standard monosaccharide (fucose, fructose, arabinose, mannose, galactose, xylose, glucose, ribose, galacturonic acid, and glucuronic acid) were acquired from Shanghai Lanji Technology Development Company. The assay kits for malonic dialdehyde (MDA), lactate dehydrogenase (LDH), superoxide dismutase (SOD) and catalase (CAT) were purchased from Nanjing Jiancheng Bioengineering Institute (Nanjing, China). Ascorbic acid (Vc), Butylated hydroxytoluene (BHT), 6-catboxy-2′,7′-dichloro dihydrofluorescein (CDCFH), 3-(4,5-dimethylthiazol-2-yl)-2,5-diphenyltetrazoliumbromide (MTT) and Butylated hydroxyanisole (BHA) were bought from Sigma-Aldrich (St Louis, MO,USA). 1,1-Diphenyl-2-picrylhydrazyl (DPPH) was acquired from Fluka Biochemika AG (Buchs, Switzerland). All other chemicals were of analytical grade.

### Preparation of polysaccharides

*T. hemsleyanum* roots were dried, ground into powder (mass 8 g) then added to 200 mL distilled H_2_O. Subsequently, 20 mg of cellulose was added to the mixture and stirred at 60 ℃ for 1 h controlling pH at 5.0. The sample was then filtered and centrifuged at 4000 rpm/min, for up to 15 min, and concentrated to 30 mL in a vacuum. Then, proteins were discarded with 1/4 volume of Sevag solution (n-butanol and chloroform (1:4, v/v)). The mixed solution was shaken for 30 min, and left static for 40 min, and the supernatant was collected, and flushed with nitrogen to remove n-butanol. The operation was repeated five times. Dialysis with continuously flowing H_2_O for 10 h then followed before addition of absolute ethanol to the dialysate, concentrating it to 85% then overnight refrigeration at 4 ℃. The obtained material was further centrifuged and separated, and the resulting sample was precipitated and washed using anhydrous ethanol, acetone and diethyl ether, and subsequently dried in a vacuum up to a constant weight, which lead to crude polysaccharides.

0.3 g of the crude polysaccharide extract was dissolved in 80 mL distilled H_2_O at 70 ℃ and stirred gradually for 20 min, then centrifuged at 4000r/min for 8 min. The supernatant was gently added to an ion exchange chromatography column (DEAE-52). Sodium Chloride (NaCl) additive was topped in molar ratios of 0, 0.2, 0.4, 0.5, 0.8 and 1.0 M. A sulphuric acid—phenol method was used to detect and track the changes in sugar content during the chromatography [22].

### Analysis of monosaccharide components

Ion chromatography (IC) technique was used to quantitatively identify the monosaccharide composition of the polysaccharides, according to the method of Wang et al., with minor modifications [23]. This was done on a Dionex ICS 5000 chromatographic system (CA, USA). 10 mg of sample was dissolved in 4.0 mL of distilled H_2_O and topped up with 0.9 mL of trifluoroacetic acid (TFA). The mixture was hydrolysed at 121 ℃ at 5000 rpm for 10 min. The resulting hyhrolytes of THP were dried by evaporation at low pressure. The TFA was totally removed by washing with methanol and the dried hydrolytes were dissolved in 1.0 mL of deionized water. Temperature was maintained at 30 ℃ with an injection volume of 25 μL. A 200 mM solution of NaOH was used as an eluent, at a volumetric flow of 0.5 mL/min. Glucose, fucose, arabinose, fructose, mannose, xylose, ribose, galactose, galacturonic acid and glucuronic acid were used as references.

### Determination of molecular weight

GPC-RI-MALS was used to determine the relative molecular mass of THP, as previously described by Zhang et al. with slight modifications [24]. Polysaccharides (5.0 mg) were dissolved in 1.0 mL of 90% dimethylsulfoxide (DMSO) and incubated overnight in glass tube immersed in a water bath at 100 ℃. Absolute ethanol (3.0 mL) was added to the polysaccharide solution and mixed vigorously. The mixture was subsequently centrifuged at 1000*g* for 5 min and the supernatant was discarded. The residual pellet was rinsed twice with absolute ethanol. This was followed by addition of 3.0 mL of 0.1 M NaNO_3_, and incubation for 20 min at 121 ℃. The resulting mixture was centrifuged at 12,000*g* for 10 min. The supernatant was collected and probed by GPC-RI-MALS (DAWN HELES II, Wyatt Technology, Santa Barbra, CA, USA) method. The temperature and flow rate were kept at 60 ℃ and 0.4 mL/min respectively. The eluent (0.1 M NaNO_3_) was examined by a refractive index detector (RI, Optilab T-rEX), with analytic column, Ohpak SB-803, 804 and 805 HQ (Shodex, Asahipak, Tokyo, Japan). Injection sample volume (100 μL) was viewed and analyzed by mass spectra furnished with ASTRA 6.1 sofware (Wyatt Technology Corpotation, Santa Barbara, CA, USA).

### Fourier transform infrared spectroscopy (FT-IR) analysis

FT-IR (Niolet AVATAR 370, US) was used to analyze the functional chemistry of THP. Briefly, 2 mg of THP was ground with standard KBr powder and subsequently condensed into pellets before recorded on the FT-IR spectrometer at a frequency ranging from 4000 to 400 cm^−1^ (Mid-infrared region).

### Congo red test

The Congo red staining assay was done according to literature [25]. Essentially, 1 mg/mL sample solution of polysaccharides, 80 μM Congo red solution and sodium hydroxide solution with different concentrations (0, 0.1, 0.2, 0.3, 0.4, 0.5, and 0.6 M) were prepared. Subsequently, sample solution (1.0 mL), Congo red solution (1.0 mL), and sodium hydroxide solution (1.0 mL) were mixed thoroughly and stored for 15 min at room temperature. The optimized absorption wavelength of Congo red in different concentrations of sodium hydroxide solution was determined by spectral scanning at 400–600 nm.

### Antioxidant activity assays

The superoxide anion radical scavenging capability was assessed by the PMS-NADH superoxide generating system [26]. The DPPH free radical scavenging activity was evaluated according to the method followed by Kamble et al. [27]. The hydroxyl radical scavenging activity was done according to the method described by Kiplimo et al. [28].

The polysaccharides antioxidant activity was also conducted by measuring the peroxidation percentage inhibition by employing the β-carotene bleaching test in linoleic acid system [29]. An emulsion of β-carotene/linoleic acid was prepared by blending 0.5 mg of β-carotene in 1.0 mL of chloroform with Tween 40 (200 μL) and linoleic acid (25 μL). The chloroform escaped entirely in a rotator at 40 ℃ under vacuum. Subsequently, distilled (100 mL) water was added and the mixture was stirred vigorously. Freshly prepared 2.5 mL aliquots of the β-carotene /linoleic acid emulsion were transferred to the test tubes with different THP concentrations (0.1–1.6 mg/mL) diluted in methanol and incubated at 50 ℃ for 60 min. The same process was repeated using butylated hydroxyanisole (BHA) as a positive standard. The absorbances of the mixtures were measured at 470 nm. The relative antioxidant activity was calculated as follows:

$$ {\text{scavenging activity (\% )}} = 1 - \frac{{{\text{A}}_{{0}} - {\text{A}}_{{{60}}} }}{{{\text{A}}_{{0}}^{{0}} - {\text{A}}_{{6{0}}}^{{0}} }} \times 100 $$

where A_0_ is the absorbance at start of incubation with THP; A_60_ is the absorbance after incubation with the THP at 60 min; ${\text{A}}_{{0}}^{{0}}$ is the absorbance at the start of the incubation without THP and ${\text{A}}_{{6{0}}}^{{0}}$ is the absorbance after incubation without THP at 60 min.

The metal chelating activity of THP was assessed according to the method described by Li et al., with some modifications [30]. Different concentrations of the sample (0.2, 0.4, 0.6, 0.8, 1.0, 2.0, or 3.0 mg/mL, 1.0 mL) were mixed with 0.1 mL of ferrous chloride (2 mM) and 3.7 mL of methanol. Reaction initiation was triggered by the addition of 0.2 mL of ferrozine (5.0 mM). The absorbance of the mixture was determined after 10 min under room temperature at 562 nm. EDTA was used as the positive control and the chelating ability was calculated as follows:

$$ {\text{chelating ability (\% )}} = \left( {1 - \frac{{{\text{A}}_{{\text{s}}} }}{{{\text{A}}_{{\text{c}}} }}} \right) \times 100\% $$

where A_c_ denotes the absorbance of the control and A_s_ represents the absorbance in the presence of the sample extracts and standard.

### Cell culture

Murine macrophage RAW264.7 cells were purchased from the Cell Bank of Type Culture Collection of the Chinese Academy of Sciences (Shanghai, China). The cells were maintained in DMEM, supplemented with 10% fetal bovine serum. The culture conditions of cells were an atmosphere of 5% CO_2_ at 37 °C.

### Detection of cell viability and cellular oxidative stress in vitro

To determine the effect of THP in hydrogen peroxide-induced oxidative stress in RAW 264.7 cells, the viability and intracellular ROS in the cells were measured. The cells were seeded and incubated for 12 h. The cells were treated with 125, 250, and 500 μg/mL of THP. After 8 h incubation, the cells were treated with 150 μM H_2_O_2_ for 3 h. The viability and intracellular ROS of hydrogen peroxide-treated RAW 264.7 cells were measured by the MTT assay and CDCFH assay, according to the method described by Ye et al. [31].

### Determination of the content of MDA and the activity of antioxidant enzyme

Cells were seeded into a 6-well plate at a concentration of 3 × 10^4^ cells/well and treated the same as “Detection of cell viability and cellular oxidative stress in vitro”. Subsequently, the cell lysate or cell supernatant was collected to assess LDH, SOD, CAT and MDA. All procedures completely followed the manufacturer's instructions.

### Statistical analysis

The data was recorded as the mean ± SD (n = 3). GraphPad Prism software (version 3.03) was utilized on the significance of differences evaluations between groups. Comparisons amongst groups were performed using the Kruskal–Wallis test ensued by Dunn’s post hoc test where the level of significance was set at P < 0.05. The IC_50_ (antioxidant concentration at which 50% of reaction was inhibited) was conducted employing the statistics program SPSS (version 18.0).

## Results and discussion

### Monosaccharide composition

**Fig. 1:**
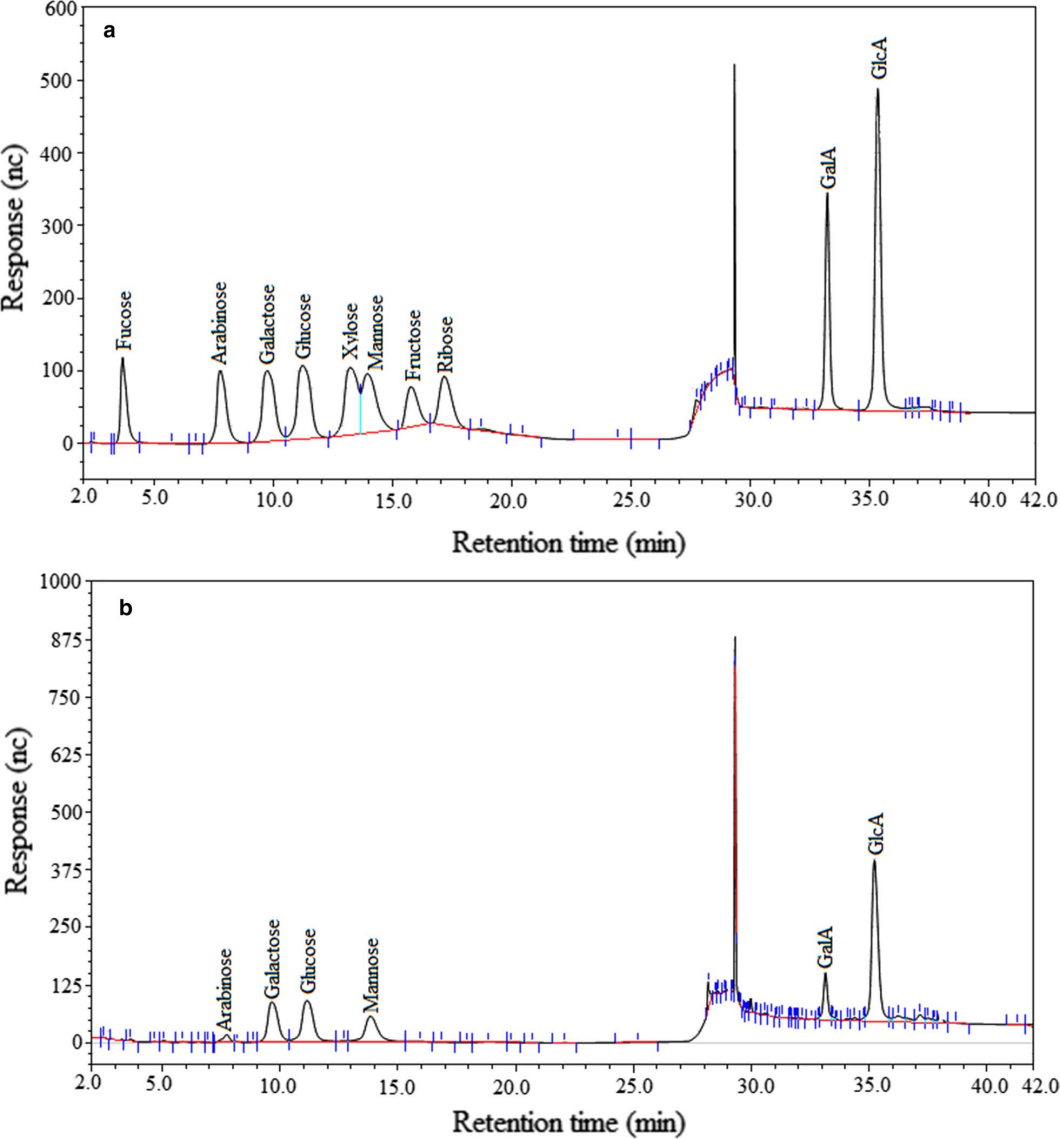
The IC chromatograms of the standard (**a**) and sample (**b**)

The ion chromatography (IC) technique was utilized to evaluate monosaccharide composition of the polysaccharides and the results were presented in Fig. 1. As clearly observed (Fig. 1 a, b), THP contained six kinds of monosaccharides in the form of arabinose, galactose, glucose, mannose, galacturonic acid, and glucuronic acid with mole ratios of 0.025, 0.159, 0.162, 0.118, 0.082, and 0.25 respectively. Glucuronic acid showed the highest content of 31.41%, indicating that the purified polysaccharides are mainly acidic polysaccharose. Monosaccharide compositions of THP are different from those of the polysaccharides extracted from the cane leaves of *T. hemsleyanum* [32]. This result indicated that the components of monosaccharide may be related to the parts of *T. hemsleyanum*.

### Determination of molecular weight

The polysaccharides molecular weight is distributed in a range, and generally presented as a mean value as shown in Table 1 for THP. M_w_, M_n_, and M_z_ of THP were computed to be 242.4 kDa, 186.3 kDa, and 1601.7 kDa, respectively. The polydispersity coefficient was 1.301 (M_w_/M_n_) or 8.587 (M_z_/M_n_). Furthermore, weight-average radius (R_w_), number-average radius (R_n_), and Z-average radius (R_z_) of THP were evaluated simultaneously and found to be 33.1, 31.3, and 44.7 respectively.

**Table 1:** The molecular weight, polydispersity, and radius mean square of THP

Item	Values
M_w_	177.1 ± 1.8
Molar mass (kDa) M_n_	186.3 ± 1.9
M_z_	1601.7 ± 21.6
Polydispersity M_w_/M_n_	1.301 ± 0.031
M_z_/M_n_	8.587 ± 0.141
R_w_	33.1 ± 1.7
Radius mean square, nm R_n_	31.3 ± 1.7
R_z_	44.7 ± 1.9

### FT-IR analysis

**Fig. 2:**
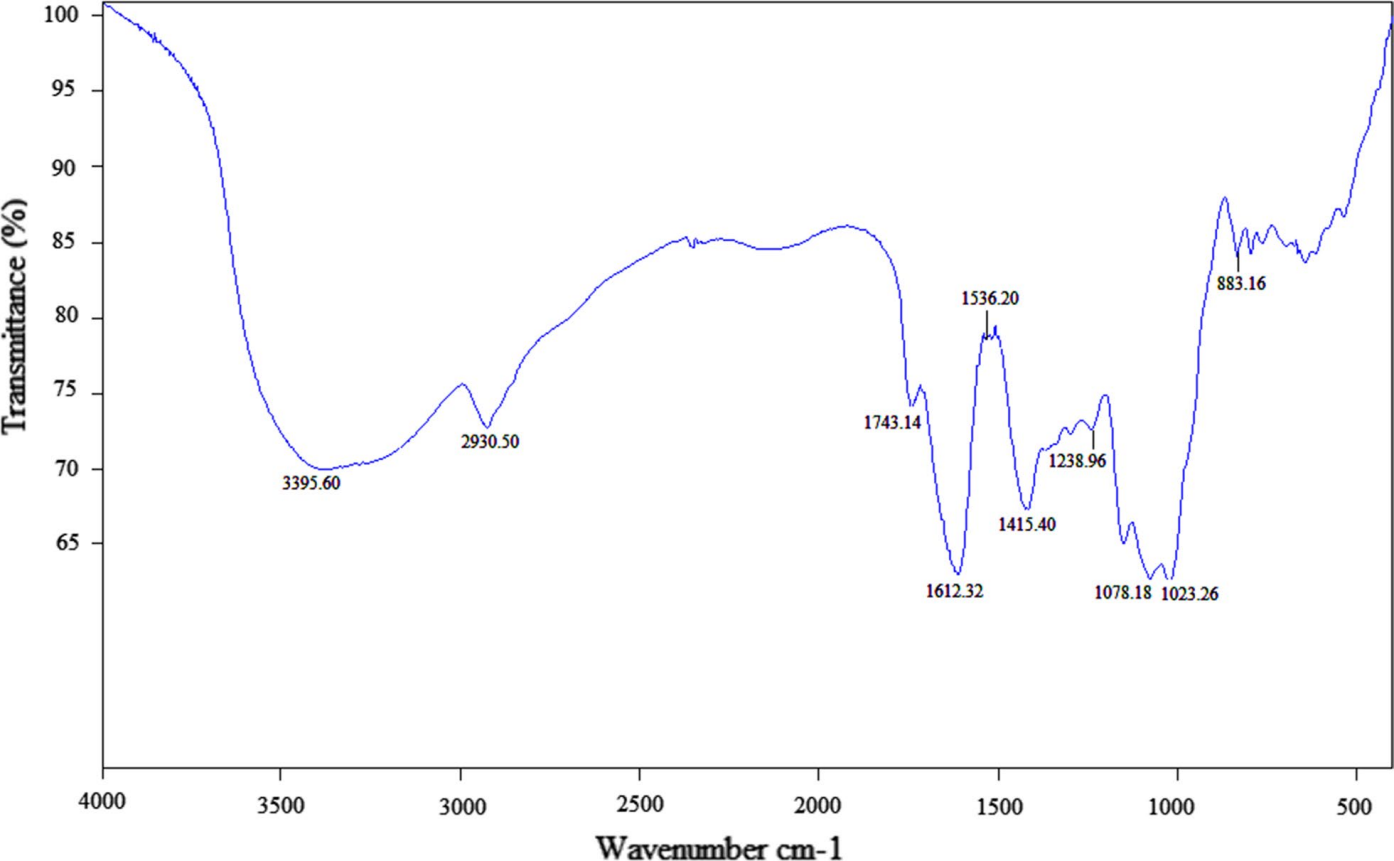
FT-IR spectroscopy of the polysaccharides

FT-IR spectroscopy, Fig. 2, was implemented to expose the key functional groups of the polysaccharides. A major wide stretching peak appearing at 3395.60 cm^−1^ was attributed to some hydroxyl functional groups, and another weak band appearing 2930.59 cm^−1^ was ascribed to some C–H stretching vibration. These two peaks characterize polysaccharides [33]. The absorption peak at 1743.14 cm^−1^ is a characteristic absorption for alduronic acid, which indicates that the polysaccharide is an acidic polysaccharose [34]. The peaks appearing at 1612.32 cm^−1^ and 1415.40 cm^−1^ were attributed to some C=O bending as well as C-H bending, respectively [35]. The peak at 1536.20 cm^−1^ was ascribed to absorption peaks of C–OH bending vibration [36]. The absorption peak appearing at 1238.96 cm^−1^ was associated with the stretching and bending vibration of hydroxyl (O–H) [37]. The characteristic absorption peaks at 1078.18 cm^−1^ and 1023.26 cm^−1^ indicated the presence of pyranose rings [38]. The weak band at 833.16 cm^−1^ was ascribed to an α-glycosidic bond in the polysaccharides framework [39].

### Conformational analysis

**Fig. 3:**
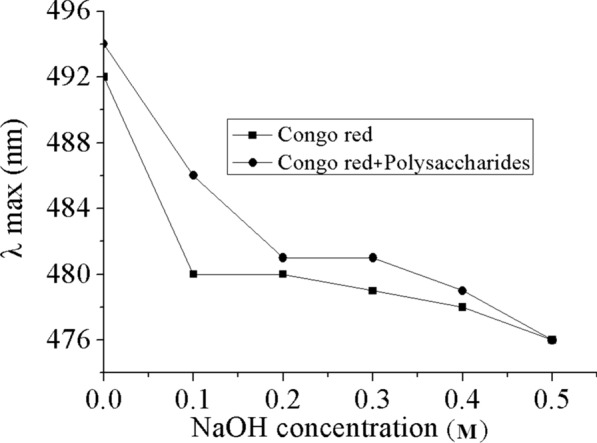
Maximum absorption wavelengths of Congo red solution and Congo red-polysaccharide complex at different NaOH concentrations

Congo red test was used to examine the triple-helix arrangements of THP and the results were displayed in Fig. 3. Congo red has the possibility of generating a complex with the polysaccharide triple-helix structure, which in turn leads to a red shift of the maximum absorption wavelength (λ _max_) due to the formed Congo red-polysaccharide complex [40]. The shifts in λ _max_ of the formed complex with varying alkaline concentration (0–0.50 M), were witnessed in THP (Fig. 3). The results showed that the maximum UV–Vis absorption wavelength of sample escalated from 480 nm in H_2_O to 486 nm in 0.1 M sodium hydroxide solution, which is an obvious indication of the presence of triple-helical assembly in THP.

### Superoxide radical scavenging activity

**Fig. 4:**
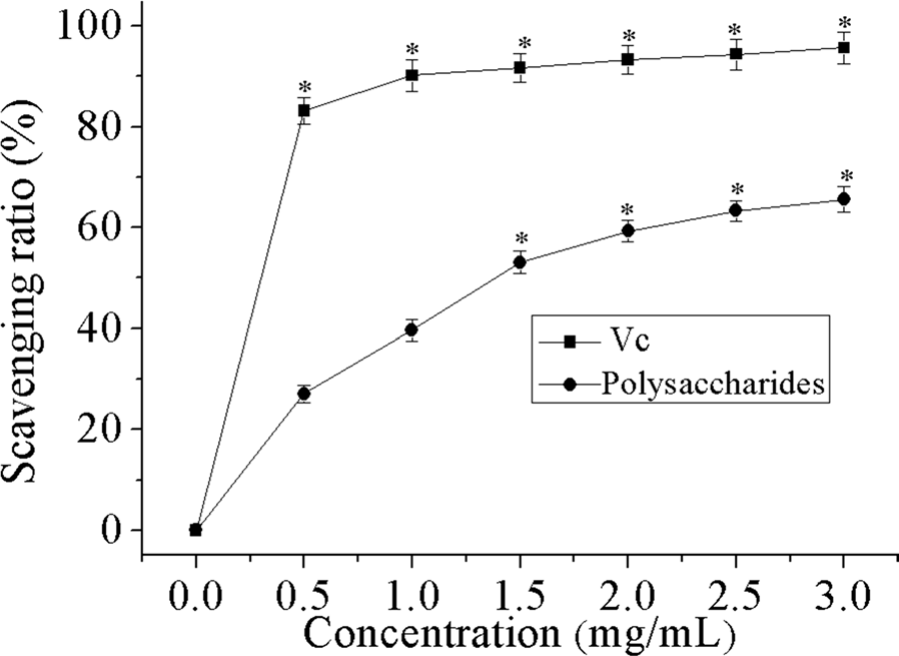
Superoxide radical scavenging activity of THP and Vc. Data comprise mean ± SD (n = 3). *P < 0.05 compared with control

Superoxide radicals are compounds formed during the metabolic activities of living organisms. An excess formation of these free radicals could break the balance and stimulate the development of various ailments which includes Alzheimer’s disease and arthritis among several others. Therefore, it is very critical to eliminate hydroxyl and superoxide radicals in organisms [41]. As can be clearly observed in Fig. 4, THP exhibited some concentration-dependent free radical scavenging effects. The maximum scavenging rate for THP (3.0 mg/mL) was perceived at 65.6%, even though still lower than that of standard V_C_ (95.7%) at the same dose. The results confirmed that the polysaccharides can exhibit some notable superoxide radical scavenging activity.

### DPPH radical scavenging activity

DPPH is a steady free radical whose ethanol solution shows an optimum absorbance at 517 nm. This absorbance is reduced when the DPPH radical encounters radical-scavenging substance (antioxidants) [42]. Thus, DPPH has been broadly utilized to determine free radical-scavenging activities of various antioxidants.

**Fig. 5:**
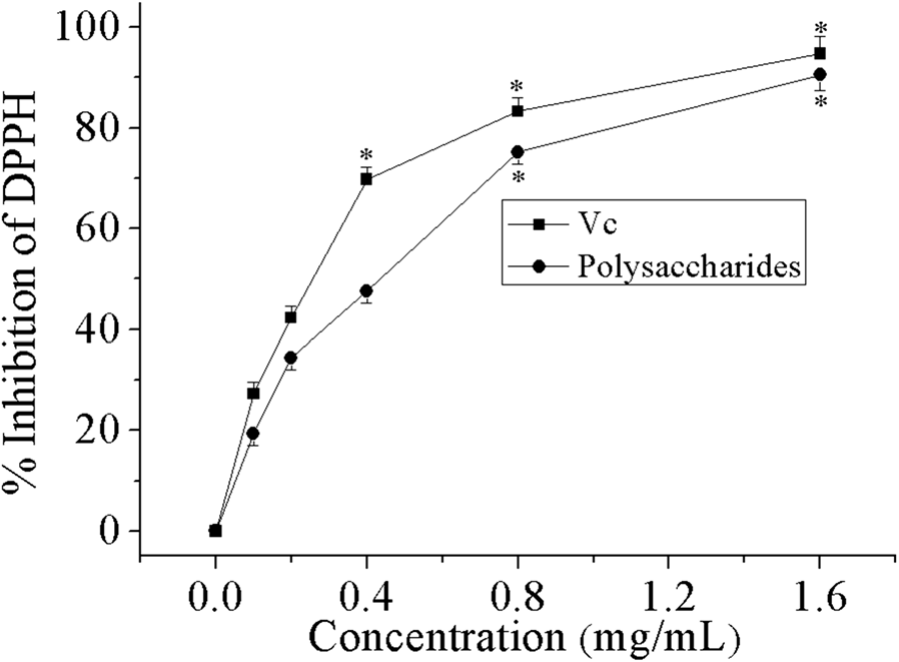
DPPH radical scavenging activity of THP and Vc. Data comprise mean ± SD (n = 3). *P < 0.05 compared with control

The result of DPPH free radical-scavenging capability of THP compared with Vc as control is displayed in Fig. 5. The DPPH radical scavenging activity escalated from 19.4 to 90.6%, when the concentration of THP was lifted from 0.1 to 1.6 mg/mL. The IC_50_ of THP and Vc were 0.43 and 0.26 mg/mL, respectively. The results specified that both THP and Vc had considerable DPPH radical scavenging potential.

### Hydroxyl radical scavenging ability

**Fig. 6:**
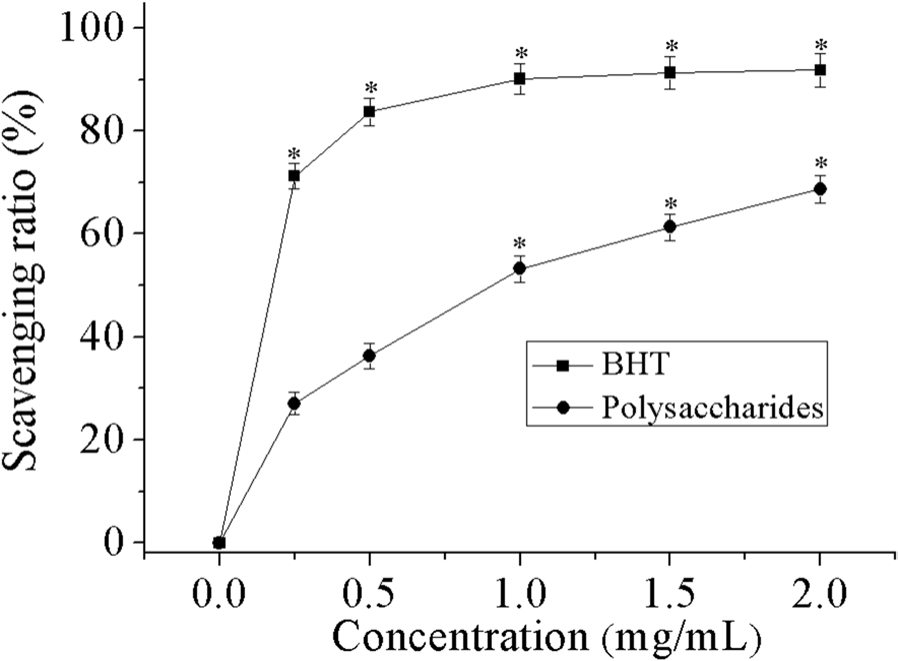
Hydroxyl radical scavenging activity of THP and BHT. Data comprise mean ± SD (n = 3). *P < 0.05 compared with control

Hydroxyl radical is reflected as greatly potent oxidants, with ability to react with all functioning bio-macromolecules in living cells, except for superoxide radicals [43]. The results for polysaccharides effect on deoxyribose degradation in comparison to BHT are presented in Fig. 6. The anti-radical activities of the studied polysaccharides and BHT at 2.0 mg/mL were 68.7% and 91.8%, respectively. The results disclosed that the scavenging capability of THP against hydroxyl radical was less than that of BHT, which is known to be a stronger hydroxyl radical scavenger.

### Inhibition of β-carotene bleaching

β-carotene undergoes vigorous discoloration in the deficiency of an antioxidant, resulting in a decline in the absorbance of the sample solution with reaction time [44]. This is because when the linoleic acid is oxidized it produces free radicals which attack the vastly unsaturated β-carotene molecules in a struggle to repossess a hydrogen atom. The presence of an antioxidant sidesteps the damage of the β-carotene thereby maintaining the featured orange color.

**Fig. 7:**
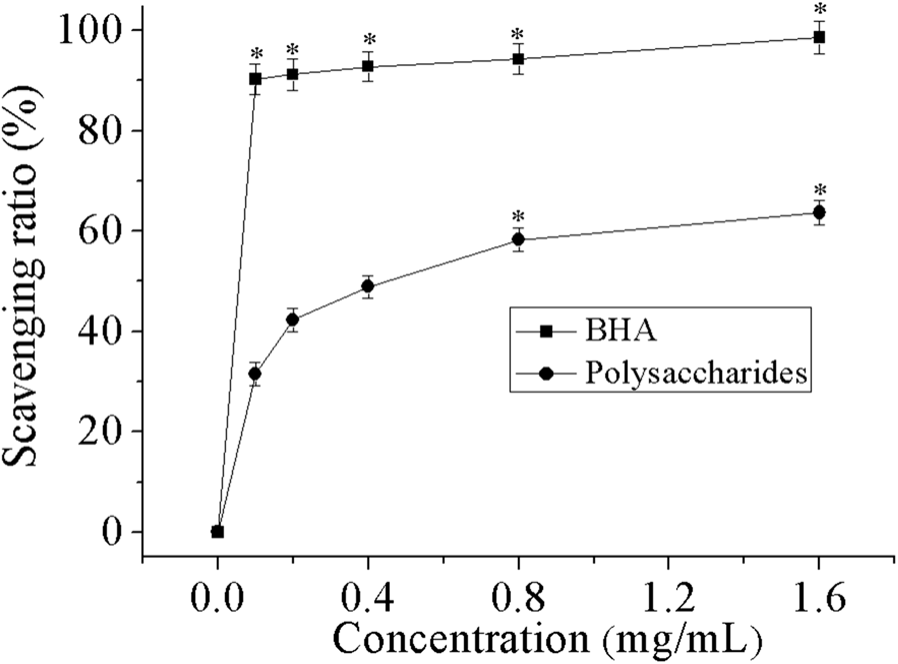
Antioxidant activity of THP and BHA using β-carotene—linoleate system. Data comprise mean ± SD (n = 3). *P < 0.05 compared with control

As shown in Fig. 7, the lipidic peroxidation inhibition percentages of THP and BHA at 1.6 mg/mL equal to 63.7% and 98.6%, respectively. The lipidic peroxidation inhibiting activity of THP escalated with the increase in concentration of polysaccharide. However, even though the inhibition activity of THP increased obviously, with increasing concentration, it remained lower than that of BHA at each dosage.

### Metal chelating ability

**Fig. 8:**
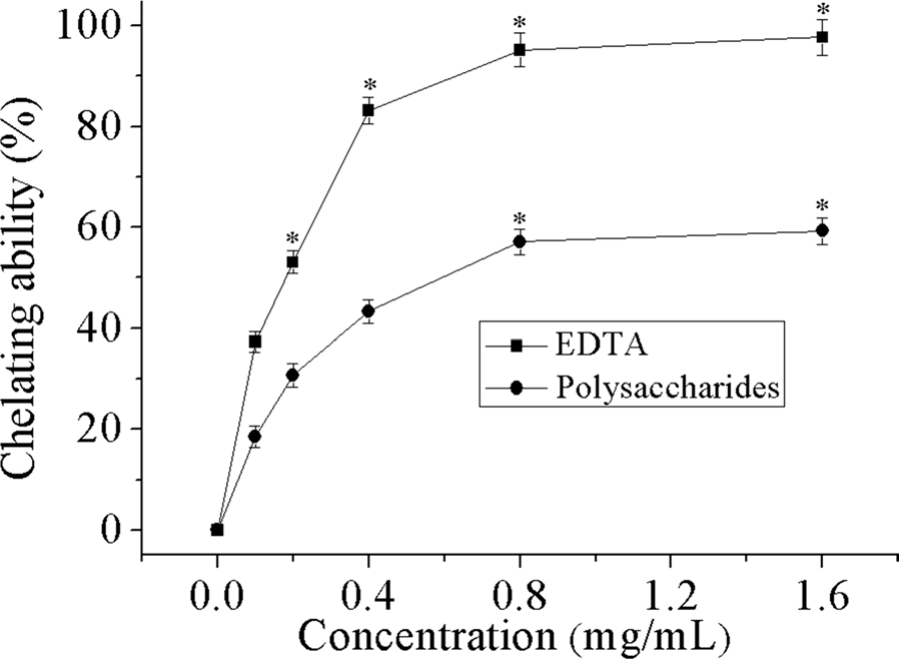
Metal chelating activity of THP and EDTA. Data comprise mean ± SD (n = 3). *P < 0.05 compared with control

Among several metal ion species, Fe^2+^ has proven to be the most potent pro-oxidant, with capacity to form complexes with ferrozine [45]. It has been demonstrated that Fe^2+^ ion speeds up lipid peroxidation by decomposing lipid peroxides and hydrogen generated by the Fenton free radical reaction [46]. Henceforth, the chelating influence on ferrous ions has been lately, a broadly used technique to assess some antioxidant activity. Figure 8 depicts the metal chelating capability of THP escalating linearly with concentrations utilized in the reaction test. Compared to EDTA, a known metal chelating agent, the chelating potency of the test polysaccharides on ferrous ion was relatively weaker. The IC_50_ value was 0.59 mg/mL for the polysaccharides and 0.18 mg/mL for EDTA.

It is acknowledged that the chelating capacity of the metal will be involved in antioxidant activity, it might, at the same time, be affecting other functionalities that contribute to the activities of anti-oxidation [47]. Hence, possibly, the polysaccharides chelating impact on Fe^2+^ might partly influence other activities of scavenging free radicals to safeguard the host organism against oxidative mutilation. Since Fe^2+^ is the most efficacious pro-oxidants in the food system, the high Fe^2+^ chelating capabilities of the polysaccharides would be somewhat beneficial [48].

### *Evaluation of the effects of THP in H*_*2*_*O*_*2*_*-stimulated RAW 264.7 cells*

**Fig. 9:**
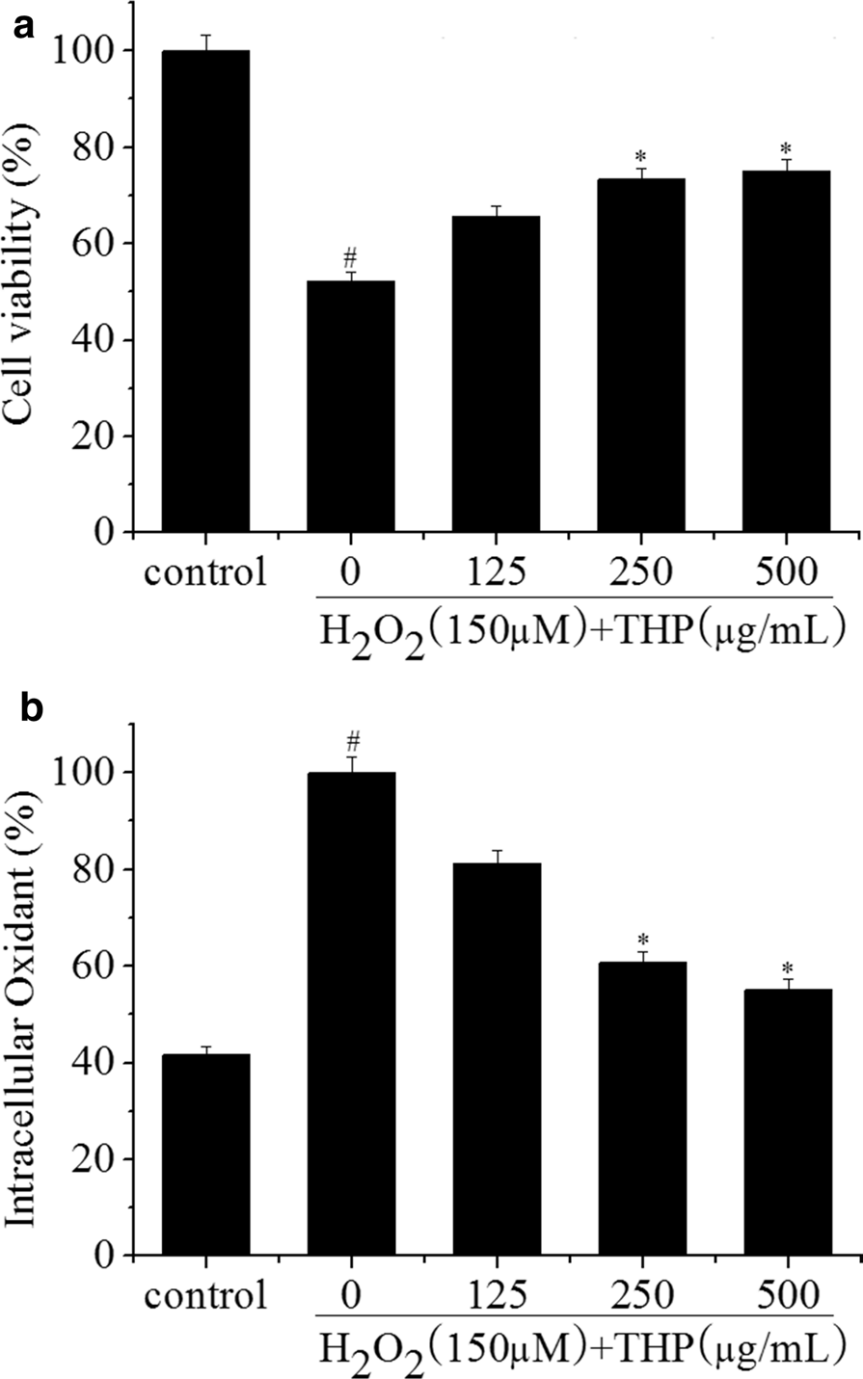
The protective effect of THP against H_2_O_2_-induced cell death in RAW 264.7 cells (**a**) and the ROS scavenging effect of THP during H_2_O_2_-induced oxidative stress in RAW 264.7 cells (**b**). Data comprise mean ± SD (n = 3). #p < 0.05 between control group and zero group. *p < 0.05 between zero group and THP pretreatment group

The cytoprotective effect of THP was detected by measuring the viability of the H_2_O_2_-treated RAW 264.7 cells. In Fig. 9a, the viability of cells exposed to 150 μM H_2_O_2_ for 3 h without THP pretreatment was 52.3% of the control value (100%), whereas the viabilities of cells treated with 125, 250, and 500 μg/mL of THP increased to 65.8%, 73.4%, and 75.2%, respectively. Moreover, THP at these concentrations alone did not cause any apparent effects on RAW 264.7 cells (data not shown). The intracellular ROS levels in cells treated with THP were lower than the group treated with H_2_O_2_ alone (100%). The Intracellular ROS levels decreased to 55.1% at a concentration of 500 μg/mL of THP (Fig. 9b). These results were similar to those of Wang et al. [49], which indicated the cytoprotective potential of THP against H_2_O_2_-treated cellular damage and suggested that THP effectively eliminated the intracellular ROS generated by H_2_O_2_-induced in a dose dependent manner.

### Assay of the content of MDA and the activity of antioxidant enzyme

MDA is a marker of endogenous lipid peroxide [50]. According to the manufacturer's protocol, MDA was detected in the culture supernatant of cells. As shown in Table 2, treatment of RAW 264.7 cells with 150 μM H_2_O_2_ caused the increase of the intracellular MDA level (1.65 nmol/mg protein), while the treatment of cells with TPH evidently reduced the release of MDA. The MDA level was 1.42 nmol/mg protein, 1.27 nmol/mg protein and 1.13 nmol/mg protein, respectively, by the treatment with THP at 125 μg/mL, 250 μg/mL and 500 μg/mL respectively.

**Table 2:** Effects of THP on the content of MDA and the activity of SOD, CAT & LDH

Treatment	MDA (nmol/mg protein)	SOD (U/mg protein)	CAT (U/mg protein)	*LDH (U/L)*
Control	0.89 ± 0.03	18.7 ± 0.6	10.4 ± 0.4	*141.3* ± *3.5*
H_2_O_2_	1.65 ± 0.06	9.2 ± 0.4	4.7 ± 0.2	*275.8* ± *6.7*
H_2_O_2_ + DMC (125 μg/mL)	1.42 ± 0.06	14.3 ± 0.4*	6.3 ± 0.3*	*213.7* ± 5.1*
H_2_O_2_ + DMC (250 μg/mL)	1.27 ± 0.05*	15.1 ± 0.5*	6.7 ± 0.3*	*195.2* ± 4.3*
H_2_O_2_ + DMC (500 μg/mL)	1.13 ± 0.05*	15.3 ± 0.5*	7.9 ± 0.4*	*186.3* ± 3.7*

Cells were cultured in DMEM medium containing different concentrations of THP for 8 h, then the cells were treated with 150 μM H_2_O_2_ for 3 h. Data are mean ± SD (n = 3)

*P < 0.05 compared with H_2_O_2_ group

As important antioxidant enzymes, LDH, SOD and CAT play a key role in the degradation of hydrogen peroxide [51]. As shown in Table 2, the LDH activities of cells after H_2_O_2_-induced were notably increased compared with the control group. The LDH activities of cells treated with THP significantly decreased compared to cells treated with H_2_O_2_ alone. The LDH activities of cells treated with 125, 250, and 500 μg/mL of THP dropped to 213.7 U/L, 195.2 U/L, and 186.3 U/L, respectively. The SOD and CAT activities of the cells in the H_2_O_2_ group (9.2 U/mg protein and 4.7 U/mg protein, respectively) were remarkably lower than those in the control group (18.7 U/mg protein and 10.4 U/mg protein, respectively). Compared with the H_2_O_2_ group, THP significantly increased the SOD and CAT activities of the cells. With the increase in THP concentration, SOD and CAT activities of cells gradually increased (Table 2). The SOD and CAT activities increased to 15.3 U/mg protein and 7.9 U/mg protein, respectively, by the treatment with TPH at 500 μg/mL. These results indicated that RAW 264.7 cells were protected from injury induced by H_2_O_2_ with pre-incubation of THP.

## Conclusion

In this research work, the polysaccharides composition and some structural details were determined. Results from the study showed that the molecular mass of THP was 177.1 kDa and glucuronic acid was the main component of the polysaccharides (31.41%), which indicated that the polysaccharides were acidic polysaccharose. FT-IR analysis revealed an α-glycosidic bond in the polysaccharides, and the Congo red binding assay confirmed the existence of a triple helix structure. The antioxidant activities study and cytotoxicity experiments showed that THP exhibited significant antioxidant abilities and that it was able to attenuate H_2_O_2_-treated cytotoxicity in RAW 264.7 cells. These results demonstrated that polysaccharides obtained from the roots of *T. hemsleyanum* had potential value in medicine and food products.

## Data Availability

The datasets generated during and/or analysed during the current study are available from the corresponding author on reasonable request.

## Abbreviations

THP: Polysaccharides from the roots of *Tetrastigma hemsleyanum*
IC: Ion chromatography
GPC-RI-MALS: Gel permeation chromatography-refractive index-multiangle laser light scattering
FT-IR: Fourier transform infrared spectroscopy
CAT: Catalase
SOD: Superoxide dismutase
LDH: Lactate dehydrogenase
MDA: Malondialdehyde
Vc: Ascorbic acid
BHT: Butylated hydroxytoluene
CDCFH: 6-Catboxy-2′,7′-dichloro dihydrofluorescein
MTT: 3-(4,5-Dimethylthiazol-2-yl)-2,5-diphenyltetrazoliumbromide
BHA: Butylated hydroxyanisole
DPPH: 1,1-Diphenyl-2-picrylhydrazyl
TFA: Trifluoroacetic acid
